# Ultrastructural Changes Associated with Reversible Stiffening in Catch Connective Tissue of Sea Cucumbers

**DOI:** 10.1371/journal.pone.0155673

**Published:** 2016-05-18

**Authors:** Masaki Tamori, Kinji Ishida, Eri Matsuura, Katsutoshi Ogasawara, Tomohito Hanasaka, Yasuhiro Takehana, Tatsuo Motokawa, Tokuji Osawa

**Affiliations:** 1 Department of Biological Sciences, Graduate School of Bioscience and Biotechnology, Tokyo Institute of Technology, Meguro-ku, Tokyo, Japan; 2 The Center for Electron Microscopy & Bio-imaging Research, Central Research Laboratories, Iwate Medical University, Morioka, Iwate, Japan; 3 Laboratory of Microbiology, Institute of Microbial Chemistry (BIKAKEN), Shinagawa-ku, Tokyo, Japan; 4 Kyushu Nutrition Welfare University, Kitakyushu, Fukuoka, Japan; Massey University, NEW ZEALAND

## Abstract

The dermis of sea cucumbers is a catch connective tissue or a mutable collagenous tissue that shows rapid, large and reversible stiffness changes in response to stimulation. The main component of the dermis is the extracellular material composed of collagen fibrils embedded in a hydrogel of proteoglycans. The stiffness of the extracellular material determines that of the dermis. The dermis has three mechanical states: soft (S_a_), standard (S_b_) and stiff (S_c_). We studied the ultrastructural changes associated with the stiffness changes. Transverse sections of collagen fibrils in the dermis showed irregular perimeters with electron-dense protrusions or arms that cross-bridged between fibrils. The number of cross-bridges increased in stiffer dermis. The distance between the fibrils was shorter in S_c_ than that in other states, which was in accord with the previous report that water exuded from the tissue in the transition S_b_→S_c_. The ultrastructure of collagen fibrils that had been isolated from the dermis was also studied. Fibrils aggregated by tensilin, which causes the transition S_a_→S_b_ possibly through an increase in cohesive forces between fibrils, had larger diameter than those dispersed by softenin, which antagonizes the effect of tensilin. No cross-bridges were found in isolated collagen fibrils. From the present ultrastructural study we propose that three different mechanisms work together to increase the dermal stiffness. 1.Tensilin makes collagen fibrils stronger and stiffer in S_a_→S_b_ through an increase in cohesive forces between subfibrils that constituted fibrils; 2. Cross-bridging by arms caused the fibrils to be a continuous network of bundles both in S_a_→S_b_ and in S_b_→S_c_; 3. The matrix embedding the fibril network became stiffer in S_b_→S_c_, which was produced by bonding associated with water exudation.

## Introduction

Echinoderms have catch connective tissues or mutable collagenous tissues whose stiffness is neurally controlled [1], [2]. Cells are sparse in this tissue, which is occupied mostly by extracellular material whose stiffness changes are responsible for those of the tissue [3]. The extracellular material could be regarded as a hydrogel of proteoglycans in which collagen fibrils are embedded [4], [5].

In the present study we examined ultrastructural changes in the extracellular material that accompanied stiffness changes. We used the body-wall dermis of sea cucumbers, the best studied material among catch connective tissues. The merit in using this material lies in that it has three clearly different mechanical states each of whose mechanical properties have been well characterized and there are detailed studies on stimuli and conditions that produce each state. The dermis exhibits: a soft state (abbreviated as S_a_), a standard state (S_b_) in which the dermis is in a non-stimulated basal state, or a stiff state (S_c_) [6]. The stiffness increases in the order S_a_ < S_b_ < S_c_ and the energy dissipation decreases in the order S_a_ > S_b_ > S_c_, from which we might expect that S_b_ is just a transitional state between the two extremes S_a_ and S_c_. The S_b_ is, however, not simply an intermediate state. The soft state S_a_ is characterized by stress softening, in which a strain of more than 10% causes a large stiffness decrease, and S_c_ is characterized by stress-strain curves without prominent toe regions. Neither of these characteristics is found in S_b_, which suggests that different mechanisms are involved in the transition from S_a_ to S_b_ (abbreviated here as S_a_→S_b_) than in the transition S_b_→S_c_ [6]. This view was supported by the discoveries of sea-cucumber-derived proteins that invoke the respective transitions. Tensilin produces the transition S_a_→S_b_ but not the transition S_b_→S_c_; this action of tensilin is antagonized by softenin [3], [7]. The novel stiffening factor (NSF) produces the transition S_b_→S_c_ but not the transition S_a_→S_b_ [8]; the transition S_b_→S_c_ is associated with water exudation from the dermis, which is not observed in the transition S_a_→S_b_ [5]. The aim of the present study was to determine the changes in the ultrastructure of the extracellular material associated with each transition.

We examined both the whole dermis and two modified preparations: dermis whose cell membranes were disrupted by the detergent Triton X-100 (Triton-treated dermis) and suspensions of collagen fibrils isolated from the dermis. We examined these two preparations because these cell-free systems also show the state transitions that have been regarded as corresponding to those in the whole dermis. The transition that corresponds to S_a_→S_b_ is reported when tensilin is applied to dermis treated with Triton and subjected to freeze-thaw (FT) treatment, in which the dermis is frozen and thawed repetitively [3]. Tensilin makes aggregates in collagen suspensions, perhaps through its binding action on collagen fibrils [7], and such aggregates are dispersed by the application of softenin or by the FT treatment [3]. We examined whether the ultrastructural changes that were observed at the transition between S_a_ and S_b_ in the whole dermis are also found in these cell-free systems.

## Materials and Methods

### Ethics Statement

Sea cucumbers used in the present study (*Holothuria leucospilota* and *Stichopus chloronotus*) are invertebrates, thus no specific permits were required. The field studies were performed in a location that was neither privately owned nor protected. The field studies did not involve endangered or protected species.

### Animals and Tissues

Specimens of the sea cucumbers *Holothuria leucospilota* Brandt and *Stichopus chloronotus* Brandt were collected in Kin Bay, Okinawa, Japan. They were shipped to Tokyo Institute of Technology and kept in a recirculating aquarium. We used *H*. *leucospilota* except for the purification of softenin. Two individuals, animal #1 and #2, were used for electron microscopic observation. The dermal samples were obtained from the dorsal interambulacral region of the sea-cucumber body walls. The dermis of this species has no apparent separation into sublayers. We used the middle region in thickness of the dermis. Three pieces of strips were cut out from the excised dermal block. The size of the strip was 0.5 mm x 1 mm x 5 mm, and its long axis corresponded to the long axis of the animal.

### Preparation of Three Mechanical States

Each mechanical state was prepared by immersing isolated dermal strips in the following artificial seawater (ASW): S_a_ in Ca^2+^free ASW (CaFASW), S_b_ in ASW with normal composition (nASW), S_c_ in ASW containing a high concentration of K^+^ (KASW) [6]. The composition of nASW was as follows: 0.50 mol l^-1^ NaCl, 50 mmol l^-1^ MgCl_2_, 10 mmol l^-1^ KCl, 10 mmol l^-1^ CaCl_2_ and 20 mmol l^-1^ Tris-HCl, pH 8.0. In CaFASW, CaCl_2_ was replaced by 7.2 mmol l^-1^ EGTA. The KASW had the following composition: 0.40 mol l^-1^ NaCl, 110 mmol l^-1^ KCl, 50 mmol l^-1^ MgCl_2_, 10 mmol l^-1^ CaCl_2_ and 20 mmol l^-1^ Tris-HCl, pH 8.0.

**Fig 1:**
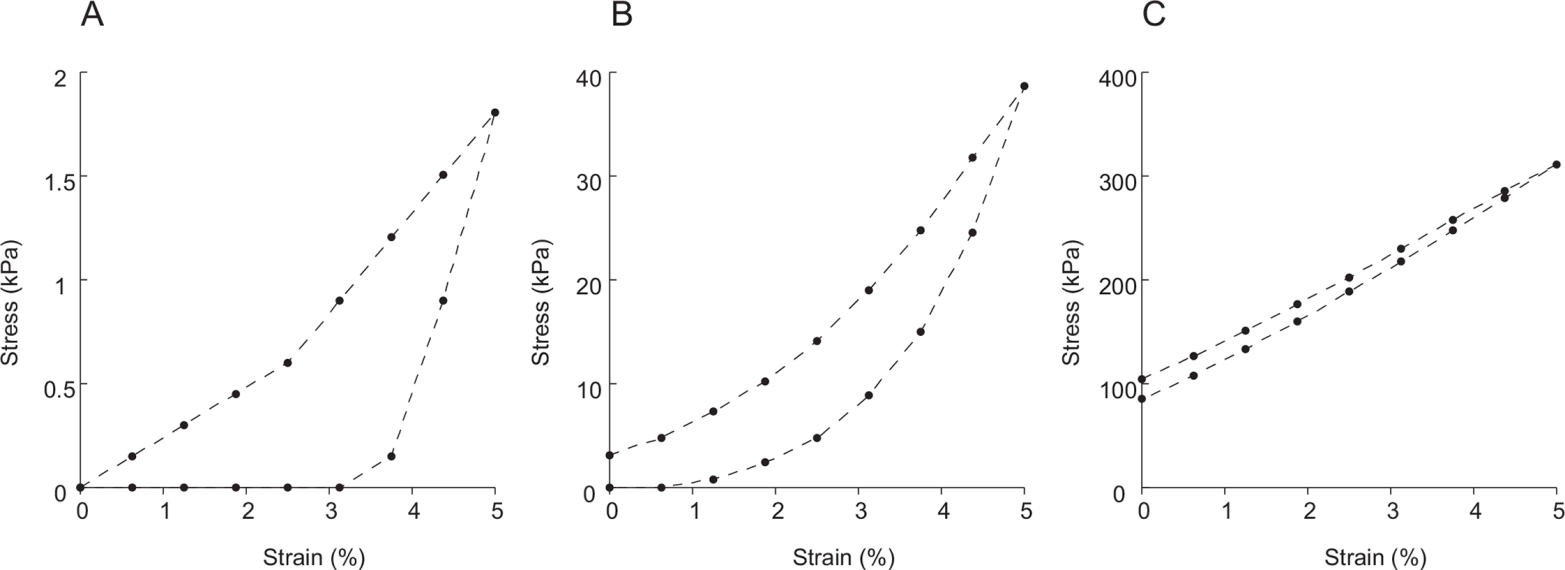
**Stress-strain curves of dermis in soft state (A), in standard state (B) and in stiff state (C).** Data from animal #1.

The isolated dermal strips were first subjected to dynamic mechanical tests to determine which mechanical state they were in. The details of the mechanical tests have been reported previously [3]. In brief, one end of the dermal strip was glued to the bottom of the trough with cyanoacrylate glue, and the other end was glued to the movable holder attached to a load cell. After the glue had cured the respective ASW was introduced to the trough. The dermal strip was immersed in the ASW for 10 minutes before mechanical testing began. Then tensile strain was applied repetitively along the long axis of the strip at a frequency of 0.3 Hz. The dermis was stretched from the original length (l_o_) to a length 5% longer than l_o_, and then it was pushed back to l_o_. The stretching generated the upper stress-strain curve and the pushing generated the lower curve (see Fig 1). The area enclosed by the two curves relative to the area under the upper curve gave the fractional energy dissipation. The stress at 5% strain divided by the strain was defined as the stiffness. The mechanical state of the sample was determined from the mechanical properties of the dermis 10 minutes after the start of the mechanical test. We supposed that the loss of macromolecules from the dermal strips was negligible in these processes. One of the reasons to suppose so was from the result of mass measurements. The mass of the dermal strips changed little after 1h soaking in nASW or CaFASW; KASW is known to induce the mass decrease but it is mainly due to water loss [5]. The other reason was the fact the isolated dermis showed stiffening and softening repetitively in response to the applications of ASW of various ionic compositions during the experiments lasted for hours [9]. Therefore we concluded that the loss of macromolecules involved in the stiffness changes was negligible, if any. The mechanical test was carried out at room temperature (23–24°C).

Triton-treated dermis was prepared by immersing the dermis in nASW containing 1% (w/v) Triton X-100 at 4°C overnight and the dermis was washed by nASW. The Triton-treated dermis was then subjected to the FT treatment to make Triton-freeze-thaw dermis (Triton-FT dermis): it was frozen and thawed ten times [3]. Dermal strips thus prepared were fixed for electron microscopy.

### Preparation of Tensilin, Softenin and Collagen Fibrils

Tensilin (*H*-tensilin) and softenin were purified from the dermis of *H*. *leucospilota* and *S*. *chloronotus*, respectively, as was reported previously [3]. Collagen fibrils were isolated from the dermis of *H*. *leucospilota* as was reported previously, including treatment with 3 mol l^-1^ guanidine-HCl and trypsin [10]. The fibrils were suspended in Tris-HCl buffer before experiments. The buffer contained 0.5 mol l^-1^ NaCl and 20 mmol l^-1^ Tris-HCl, pH 8.0. The aggregated fibrils were obtained by mixing 20 μl of suspension that contained collagen fibrils from 1.4 mg dermis with the same volume of Tris-HCl buffer containing tensilin. The final concentration of tensilin was 20 μg ml^-1^. The aggregated collagen fibrils were dispersed by adding softenin (final concentration 23.5 μg ml^-1^) or by FT treatment according to Takehana et al. (2014) [3]. The aggregated fibrils and the dispersed ones were fixed for electron microscopy.

### Electron Microscopy

The dermal strips and suspended collagen fibrils were pre-fixed in a solution containing 1.5% paraformaldehyde and 1.5% glutaraldehyde in 0.1 mol l^-1^ sodium cacodylate buffer (pH 7.4) for 1 day at room temperature. After rinsing with the buffer, the specimens were post-fixed with 1% OsO_4_ in the same buffer. The specimens were then dehydrated with a graded series of ethanol, embedded in epoxy resin and cut at 70 nm. The sections were made either along the direction of the applied tensile strain or perpendicular to it. The sections were stained with uranyl acetate followed by lead citrate and examined under a transmission electron microscope (H-7650; Hitachi, Japan) in a high contrast mode at 100 kV.

The mechanical tests that applied strain repetitively on the dermis caused collagen fibrils to align along the direction of the strain (see Results). Thus the sections cut perpendicular to the strain gave micrographs in which most fibrils showed their cross sectional views. The fibril diameter, the distance between fibrils and the number of cross-bridges connecting adjacent fibrils were measured in these micrographs. Six micrographs, three from sea cucumber #1 and three from sea cucumber #2, were used. The diameter of a fibril was the mean of the largest and the smallest diameter. The diameters of 180 fibrils were measured. The number of cross-bridges that connected one fibril to the adjacent fibrils in a micrograph was the averaged value of 30 adjoining fibrils in each electron micrograph. These mean values were again averaged to give the number of cross-bridges. The distance between fibrils was measured as follows. A randomly oriented straight line was drawn on a micrograph. The line was 1 μm-long with a midpoint that corresponded to the center of the micrograph. The line crossed several fibrils. All the distances between two adjacent fibrils on the line were measured. The results from six micrographs were combined and averaged to give the distance between fibrils. The repeat interval of the axial striation pattern of collagen fibrils (D period) was the average of four fibrils in each state. The average was the averaged value of 4–9 successive repeats in each fibril.

### Statistics

In the statistical analysis of the number of cross-bridges, the distance between fibrils and the D period of fibrils, analysis of variance was performed and *post-hoc* tests were used to test statistical differences between states. A Dunnet *post-hoc* test was used when the standard-state dermis was compared with those in other states. The difference between the mean of the soft state and that of the stiff state was statistically compared using the Bonferroni *post-hoc* test. A Steel-Dwass test was used to compare the diameter of collagen fibrils of dermis in three different states and to compare the fibril diameter of three kinds of collagen suspensions. Mann-Whitney U tests were used in the statistical analyses of the distance between fibrils and in the comparison of the diameter of fibrils between two kinds of Triton-treated dermis. A *t*-test was used to compare the means of the number of cross-bridges of the two kinds of Triton-treated dermis because these data were normally distributed.

## Results

### Mechanical Properties

The stiffness, the energy dissipation and the stress-strain curves of the dermal strips treated with three different kinds of ASW are given in Table 1 and in Fig 1. Animal #1 showed quite large stiffness changes: the stiffness decreased to 1/20 in CaFASW and it increased 8 times in KASW compared with the stiffness of the standard state S_b_ that was defined as the state in nASW [6]. Although stiffness changes were not so prominent as in #1, stiffness still halved in CaFASW and increased 4 times in animal #2. The features of the stiff state S_c_ is not only high stiffness but also low energy dissipation as well as the disappearance of the toe region in the stress-strain curves found in other states; the features of the soft state S_a_ is not only the low stiffness but also very large energy dissipation [6]. These features were found both in animals #1 and #2. Therefore we concluded that the dermal strips in CaFASW were in S_a_ and those in KASW were in S_c_. Because we could not find differences in ultrastructural features between individuals, the numerical data given below are the pooled ones from two individuals.

**Table 1:** Stiffness of dermis (upper row, in kPa) and energy dissipation (lower row given in parenthesis, in %).

Animal	CaFASW	nASW	KASW
**#1**	**39**	**773**	**6194**
	**(67.5)**	**(43.0)**	**(6.4)**
**#2**	**259**	**484**	**2175**
	**(51.5)**	**(32.3)**	**(15.4)**

### Dermis in Standard State

**Fig 2:**
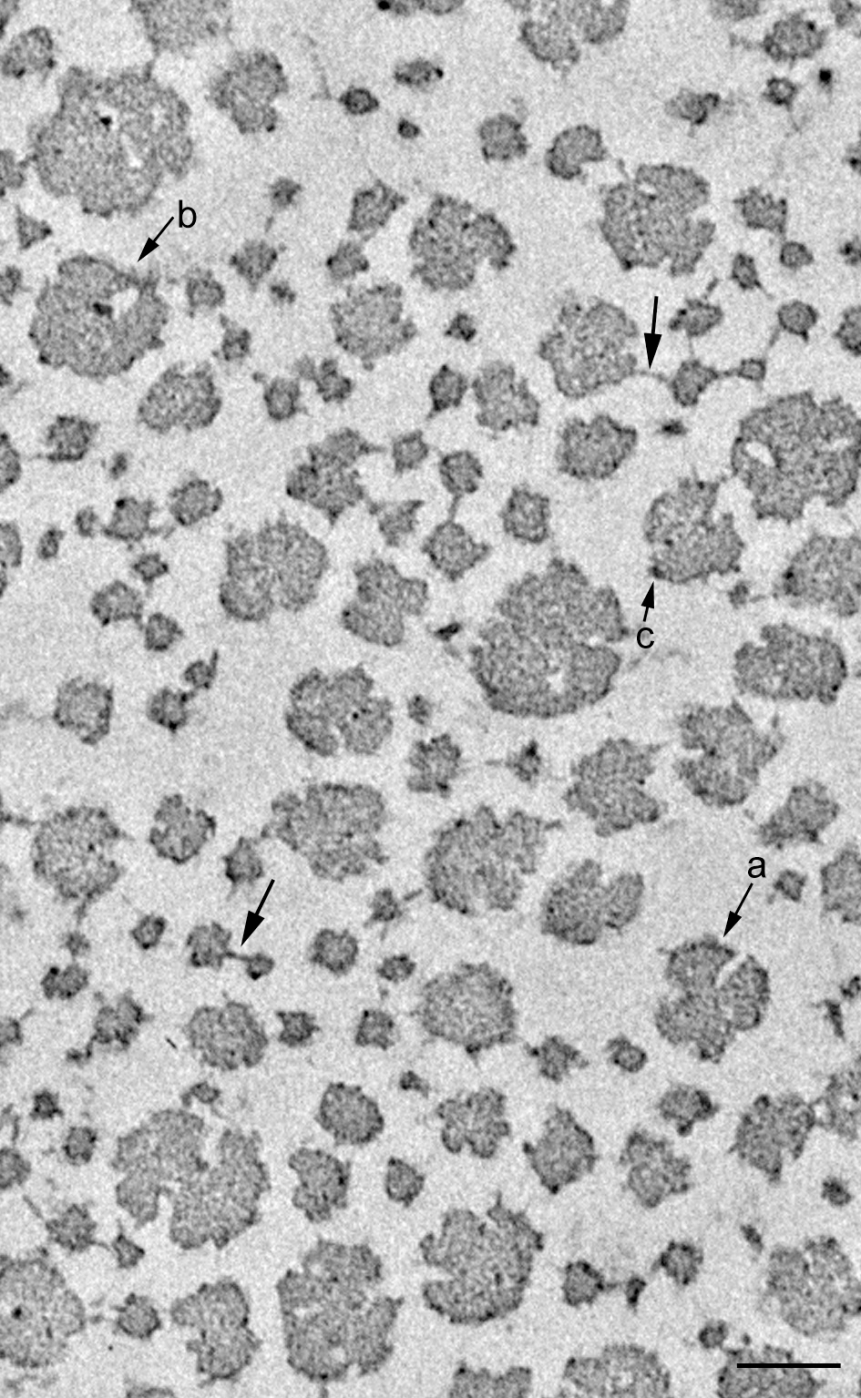
Electron micrograph of dermis in the standard state cut transversely to the strain direction. Collagen fibrils in cross sections are observed. In this, and Figs 4 and 9, unlabelled arrows denote cross-bridges between fibrils. Arrow marked a: fibril with a meandering perimeter that folds deeply towards the center of the fibril, suggesting that the fibril is possibly made of 4 subfibrils. Arrow marked b: thick fibril containing an electron-lucent area in which an electron-dense spot is observed at the border of the electron-lucent area. Arrow marked c: electron-dense spot on the perimeter of fibrils. Scale bar, 100 nm.

**Fig 3:**
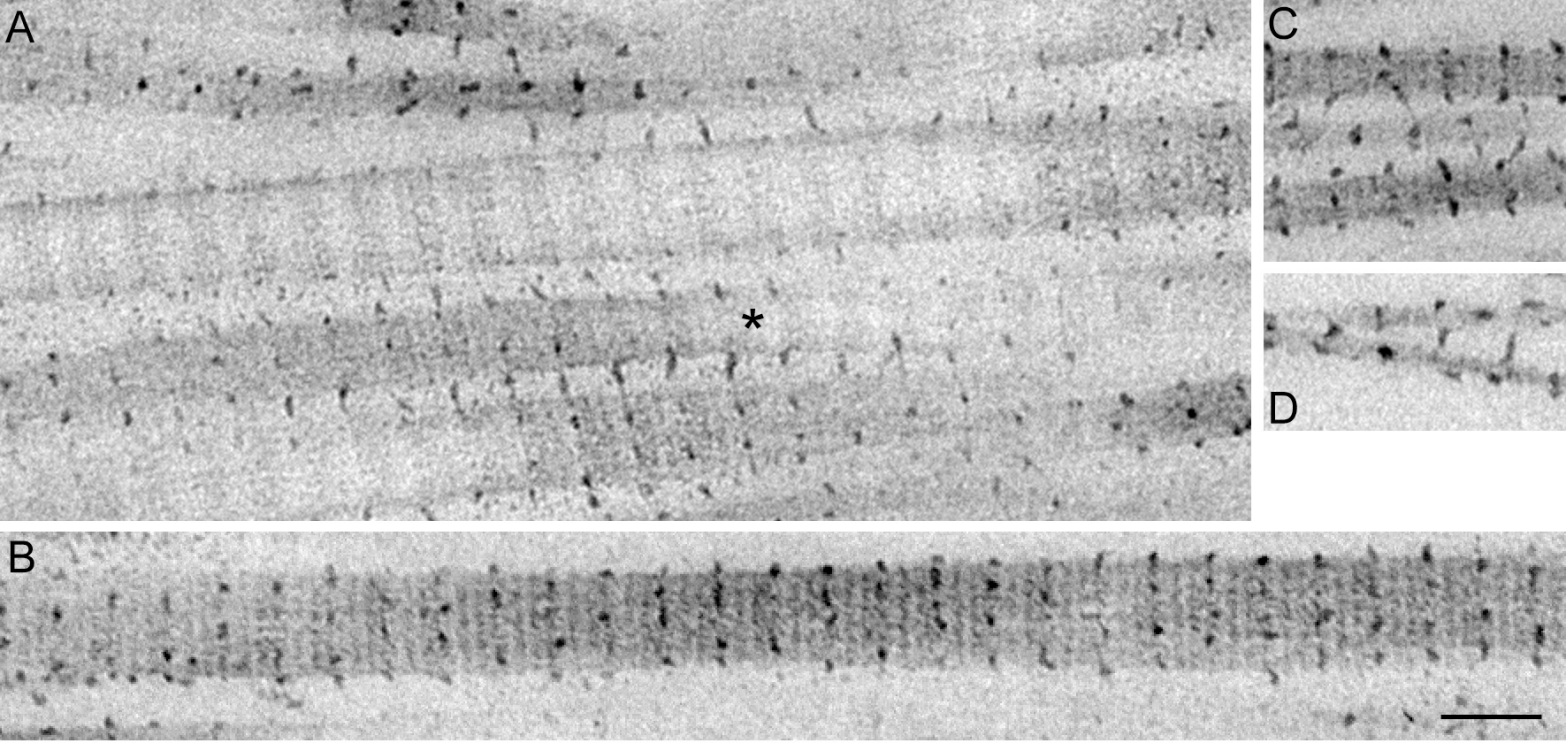
Electron micrographs of dermis in the standard state cut along the strain direction. Longitudinal sections of collagen fibrils are observed. The fibrils have protruding arms some of which make cross-bridges between fibrils. A: cross-bridges and arms that appeared repetitively along fibrils. The repeat interval was measured in the fibril marked with an asterisk. B: arrays of electron-dense spots aligned on the same plane perpendicular to the length of the fibril as that of the protruding arms. C: diagonal cross-bridges. D: cross-bridges between the fibrils that are not in parallel. Scale bar, 100 nm.

**Fig 4:**
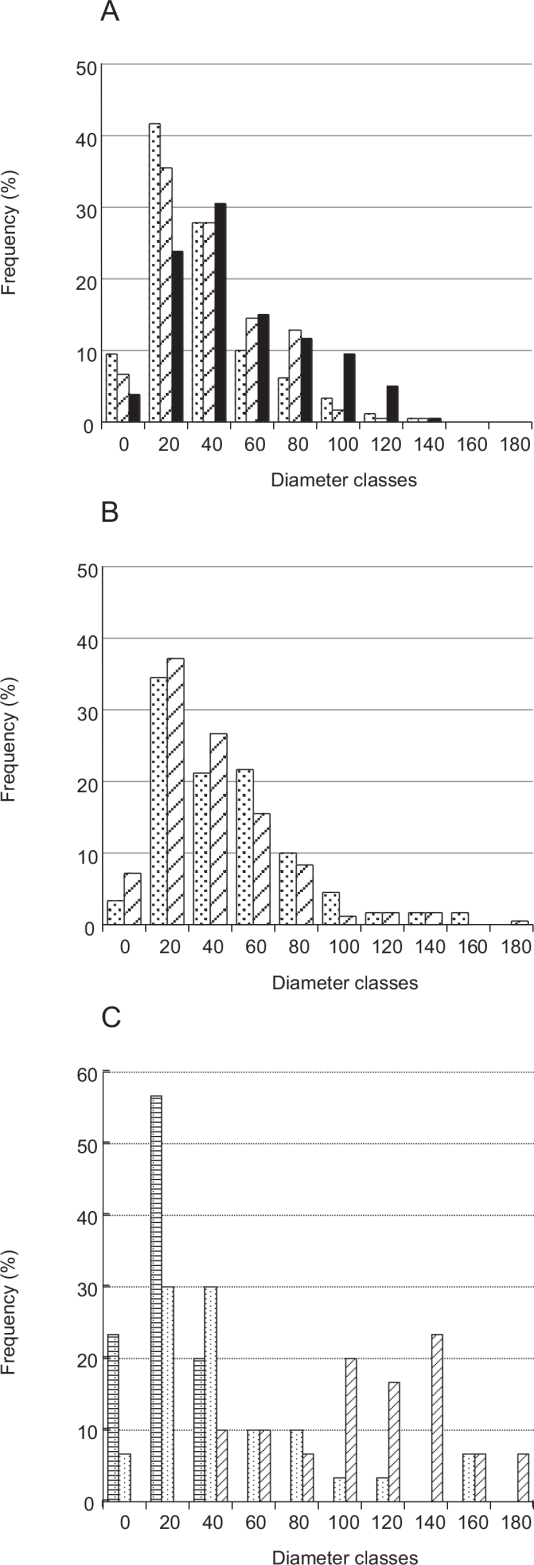
Frequency distribution of fibril diameter. A: dermis. Dermis in S_a_ (stippled bars), in S_b_ (diagonally hatched bars) and in S_c_ (filled bars) are compared. B: Dermis treated with Triton. Triton-treated dermis (diagonally hatched bars) and Triton-FT dermis (stippled bars) are compared. C: Collagen suspensions. The samples aggregated by tensilin (diagonally hatched bars), dispersed by softenin (stippled bars) or dispersed by FT-treatment (stippled bars with horizontal stripes) are compared. The diameter class 20, for example, includes the fibrils with the diameter equal to or larger than 20 nm and less than 40 nm. The total numbers of measured fibrils were: 180 for each state in A, 180 for each treatment in B, and 30 for each solution. In this and Figs 6, 7 and 8 the slant hatching and the stippling denote that the samples were in the state that mechanically corresponded to the standard state and to the soft state respectively.

Most of the collagen fibrils showed their cross sectional views in the sections cut transversely to the applied strain (Fig 2) and showed their longitudinal views in those cut along the strain axis (Fig 3) in contrast to the observation that sea-cucumber dermis is composed of three-dimensionally but not orthogonally arranged network of collagen fibrils [11]. Thus the repetitively applied strain in the mechanical tests caused collagen fibrils to align along the direction of the strain. The fibrils in the transverse sections had irregular perimeters (Fig 2). In some fibrils the perimeter deeply meandered toward the center of the fibrils. Some thick fibrils of diameter more than ca. 100 nm contained internal electron-lucent areas. The distribution of the fibril diameters had a wide range of 8 to 146 nm (Fig 4A). It was unimodal with a long tail to the right. The most frequently observe range was 20–40 nm. The mean fibril diameter was 49.8 ± 24.9 nm (average ± S.D., n = 180). The distance between fibrils was 76.8 ± 54.9 nm (average ± S.D., n = 39). The fibrils occupied 26.5±7.1% (average ± S.D., n = 6) of the area of the observed field.

Fibrils had electron dense protrusions or arms, some of which apparently made cross-bridges between adjacent fibrils (Figs 2 and 3). In a cross sectional view the number of cross-bridges per fibril was 0.46 ± 0.19 (average ± S.D., n = 6); the maximum number was 4. When multiple arms protruded each arm usually cross-bridged different neighbors but in some cases the two neighboring fibrils were connected with two bridges. The longest bridge observed was 67 nm. In a longitudinal view the arms appeared repetitively along the longitudinal axis of the fibrils with the interval that almost corresponded to the D period of collagen: for example the fibril shown in Fig 3A had a repeat interval of the arms equal to 55.8 nm and the D period was 55.7 nm. Spots with a similar electron density as that of the arms appeared on the same plane perpendicular to the length of the fibrils as that of the protruding arms (Fig 3B). The position of these spots and arms looked as if it had corresponded to a particular zone of the D-periodic cross-striation, although the resolution of our photos was not high enough to specify the zone. The D-repeat patterns of adjacent fibrils were usually aligned. Thus, lines of arms and electron-dense spots were aligned in adjacent fibrils. In such cases, the cross-bridges protruded perpendicularly to the fibril, connecting the aligned lines of spots (Fig 3A). When the lines of spots of the adjacent fibrils were not aligned, the cross-bridges were slanted with respect to the longitudinal axis of the fibrils (Fig 3C). When the adjacent fibrils were not parallel, the length of cross-bridges increased as the distance between two adjacent fibrils increased, suggesting that the arms were extensible (Fig 3D). Spots with similar electron density to that of the arms were observed on the periphery of fibrils in transverse sections (Fig 2). Some of these spots, as well as those observed on the fibrils in the longitudinal sections, possibly corresponded to arms protruding out of the plane of the section or to arms not protruded but folded. Spots with similar electron density were sometimes observed on the contour of the electron-lucent areas in the thick collagen fibrils (Fig 2).

### Comparison of Dermis in Three States

**Fig 5:**
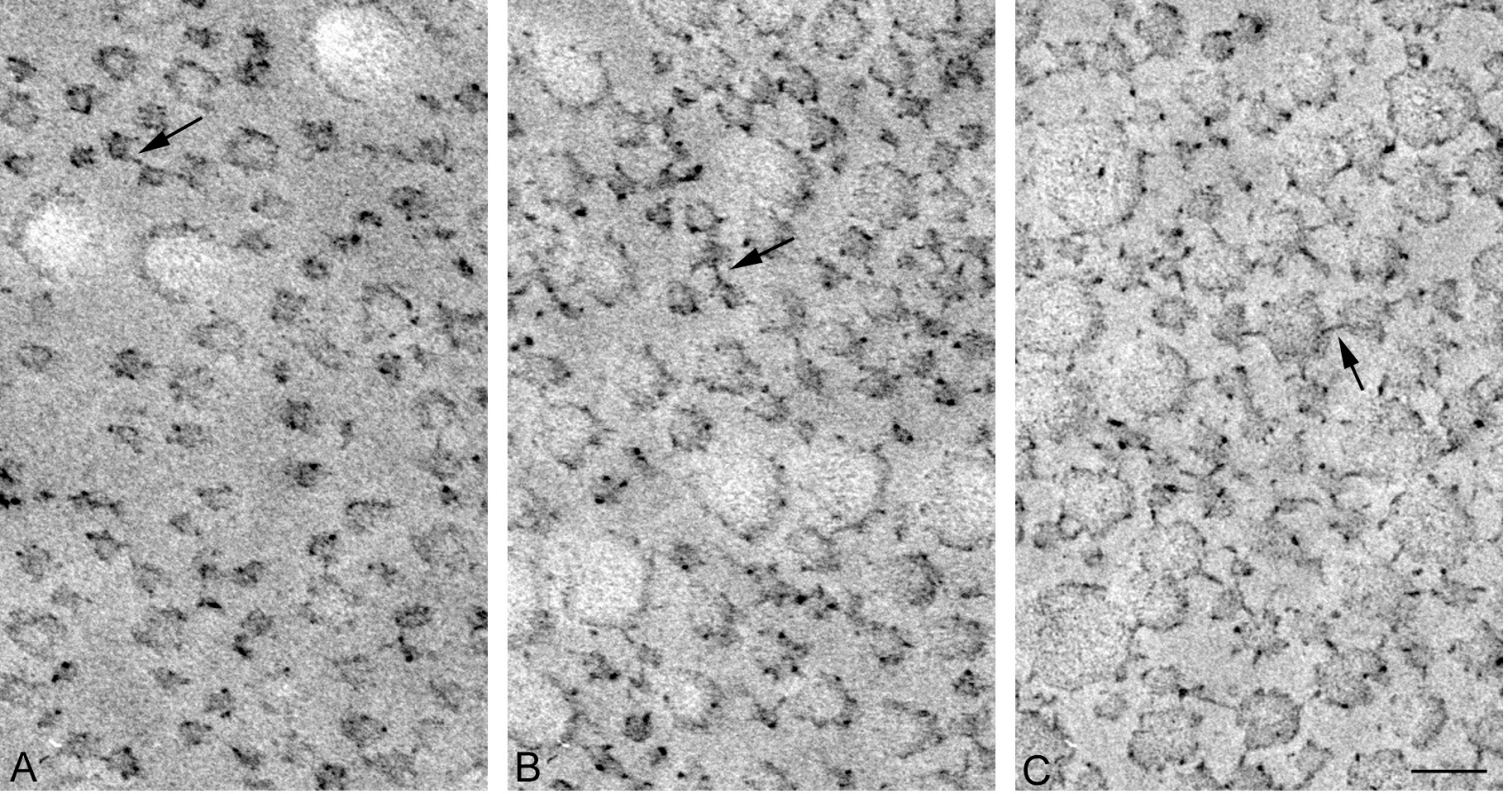
Comparison of cross sections of dermis in three different mechanical states. A, soft state; B, standard state; C, stiff state. Scale bar, 100 nm.

**Fig 6:**
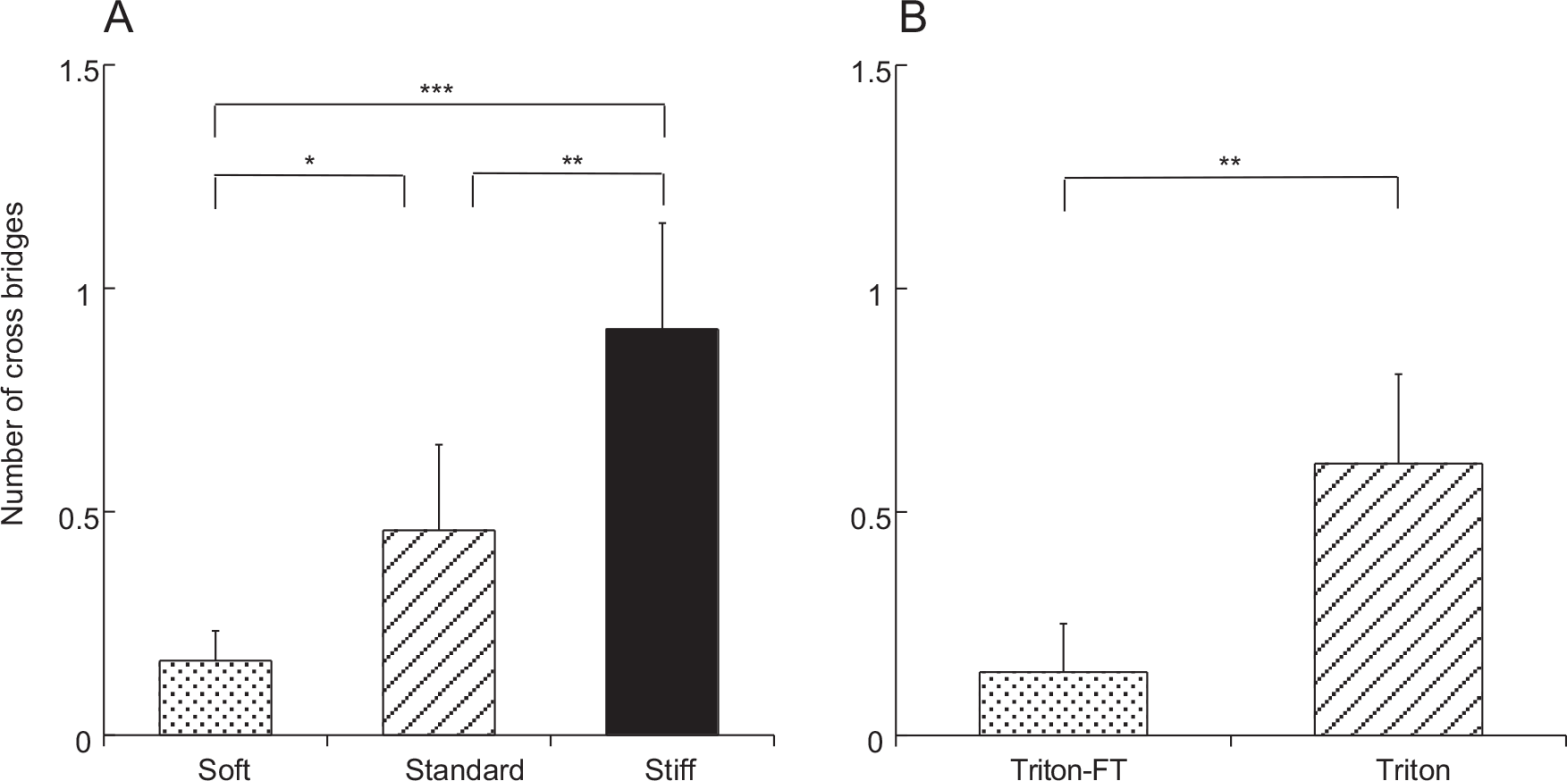
Number of cross-bridges per fibril in dermis. A: dermis in soft state, in standard state and in stiff state. B: Triton-treated dermis (Triton) and Triton-FT dermis that was treated with Triton followed by the freeze-thaw treatment. In this, and Figs 7 and 8, bars denote means; a vertical line on the bar shows +S.D.; asterisks indicate the presence of statistically significant difference (*P<0.05, **P<0.01 and ***P<0.001). The number of measurements was 6 in each state or treatment.

Transverse sections of the dermal samples from a sea cucumber fixed in three different mechanical states are shown in Fig 5. The qualitative features of collagen fibrils described above were maintained both in S_a_ and in S_b_. However, there were differences in quantities. There was a significant increase in the number of cross-bridges observed in stiffer states (Fig 6A): S_c_ had twice as many cross-bridges as S_b_, and S_b_ had about three times as many cross-bridges as S_a_. The mean numbers were statistically different between states.

**Fig 7:**
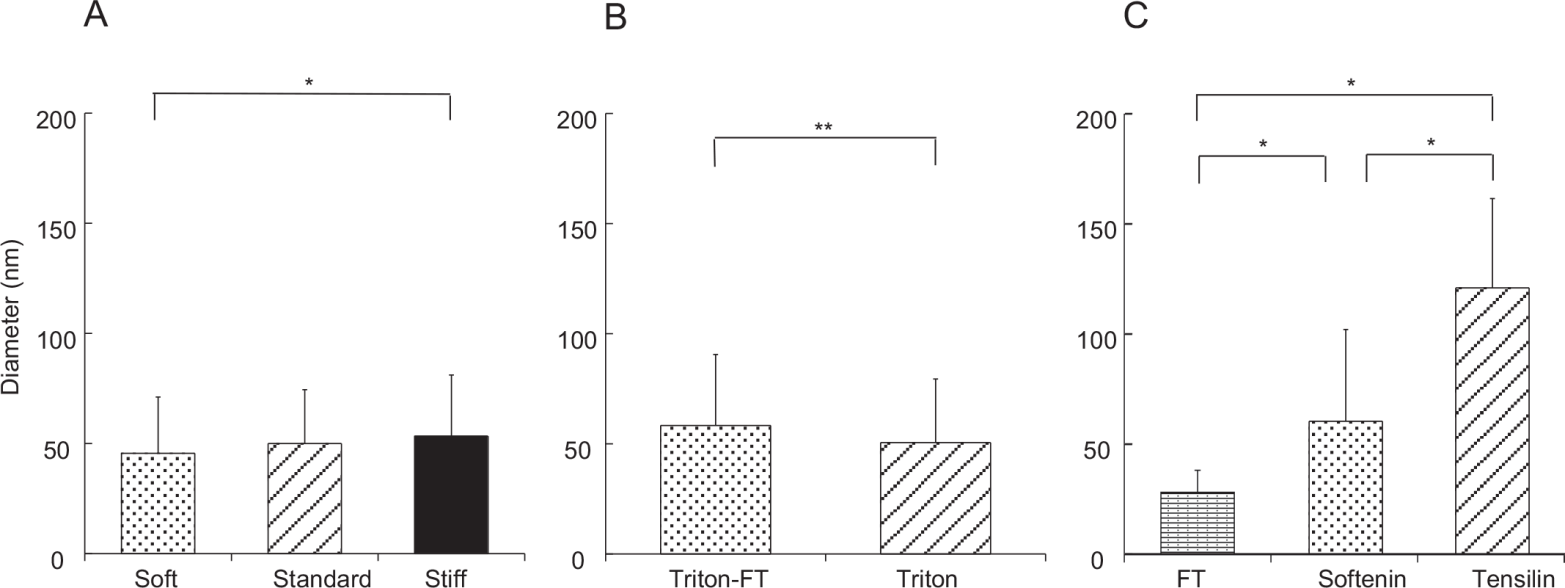
Diameter of collagen fibrils. A: dermis. Dermis in S_a_ (stippled bars), in S_b_ (slant hatched bars) and in S_c_ (filled bars) are compared. B: Triton-treated dermis (slant hatched bars) and Triton-FT (stippled bars) dermis are compared. C: collagen suspensions. The sample aggregated by tensilin, that dispersed by softenin or that dispersed by FT-treatment (FT) are compared. The number of measurements in each sample was: 180 in A and B, and 30 in C.

The distribution of fibril diameters is given in Fig 4A. Although the distribution patterns were similar in the three states, small increases in mean diameters in stiffer states were observed (Fig 7A). The mean of S_c_ was 7% larger compared to the mean of S_b_; the latter was again 7% larger than the mean of S_a_. The diameter of S_a_ and that of S_c_ were statistically different but that of S_b_ was not significantly different from those of S_a_ and S_c_.

**Fig 8:**
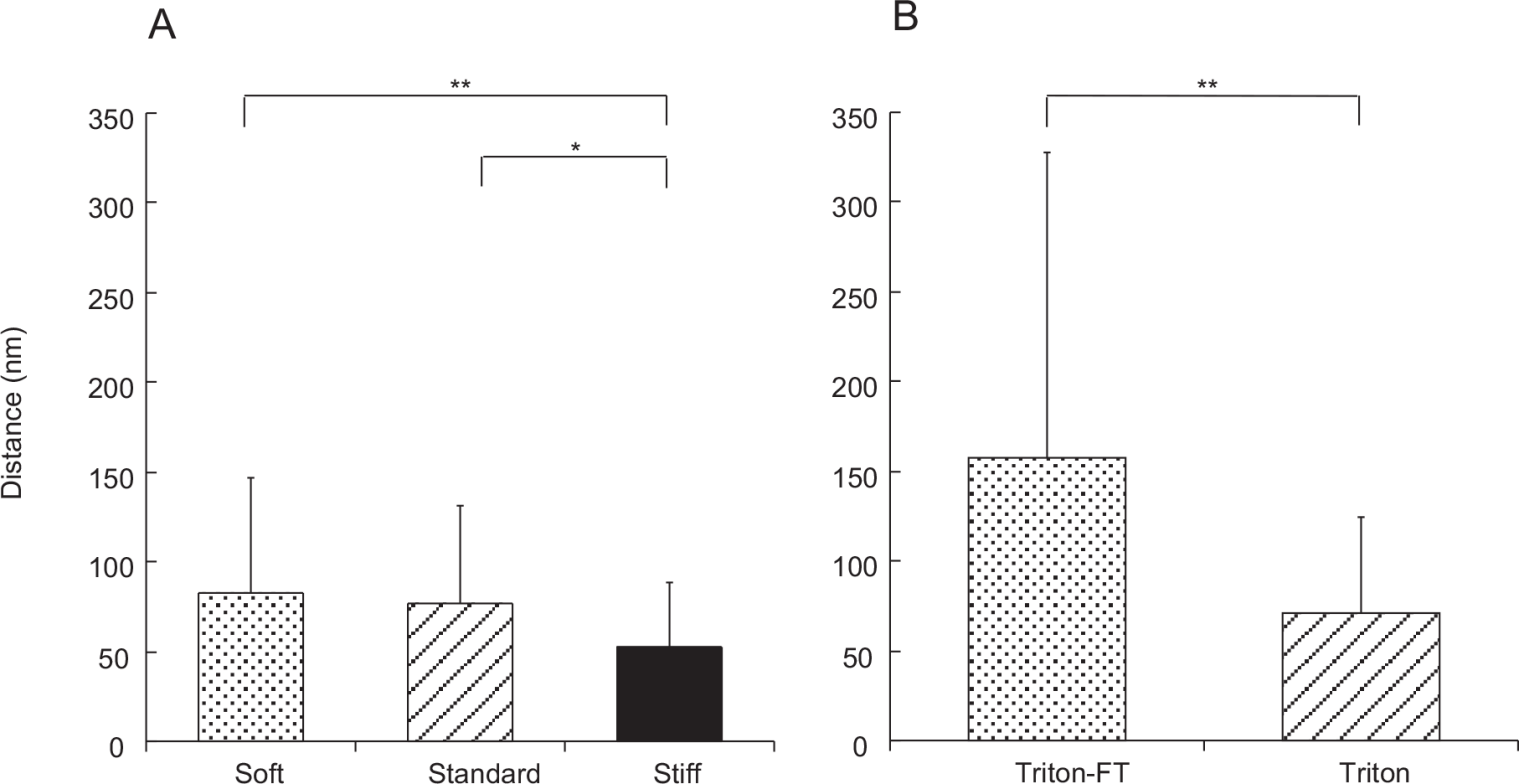
Distance between collagen fibrils. A: dermis. The dermis in S_a_, in S_b_ and in S_c_ are compared. B: Triton-treated dermis and Triton-FT dermis are compared. The numbers of measurements in A are as follows: S_a_, 41; S_b_, 39; S_c_, 62. The numbers of measurements in B are 22 for Triton-FT samples and 48 for Triton-treated samples.

A significant decrease in the distance between collagen fibrils was observed in S_c_ (Fig 8A). The mean distance in S_c_ was two thirds that in S_b_. The mean distance in S_b_ was 8% less than that of S_a_, although this difference was not statistically significant.

The D period of the dermis (average ± S.D., n = 4) was 58.3 ± 3.7 nm in S_a_, 56.2 ± 0.8 nm in S_b_, and 58.7 ± 1.1 nm in S_c_. There were no statistical differences between the averages.

### Triton-Treated Dermis

**Fig 9:**
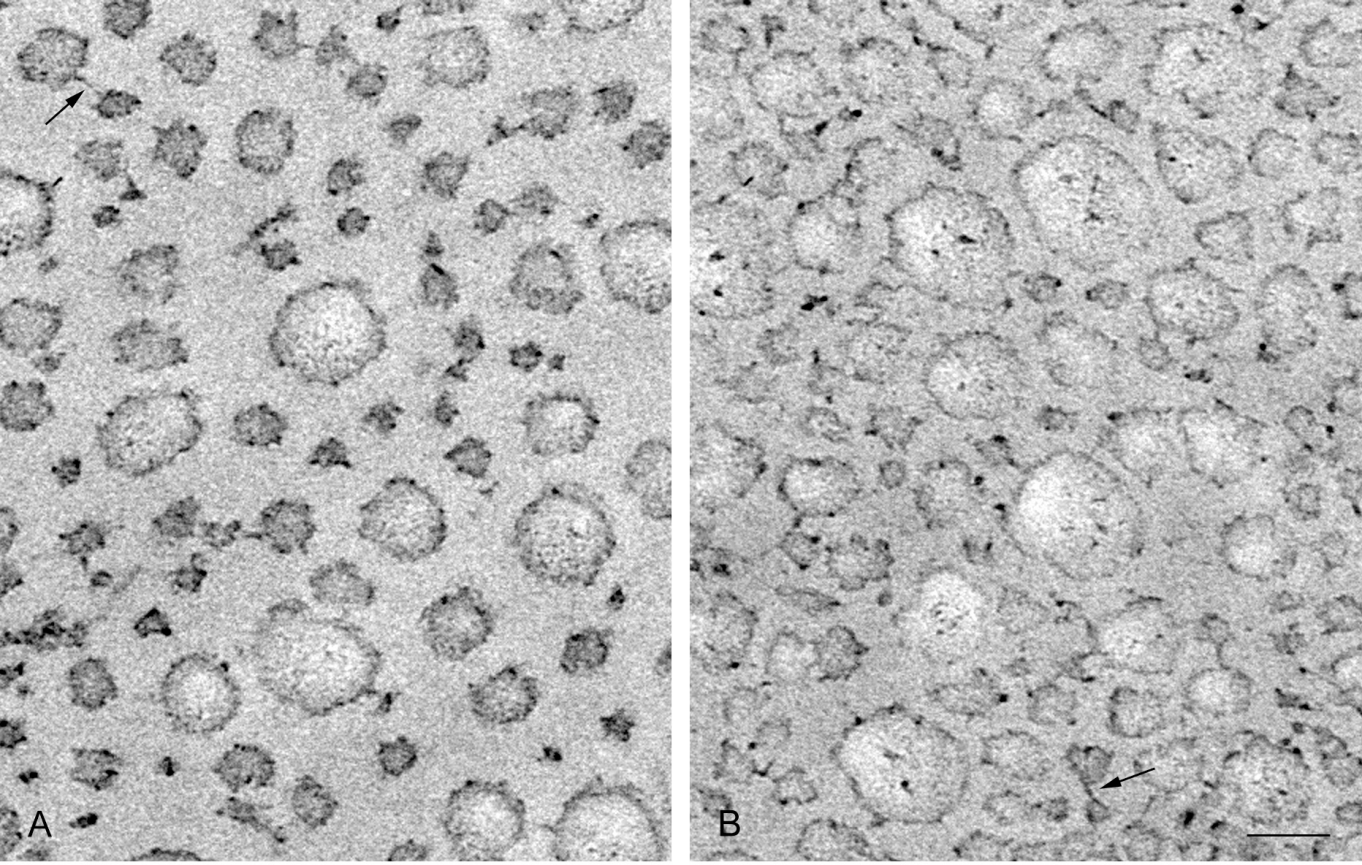
Electron micrographs of dermis treated with Triton. A: Triton-FT dermis; B: Triton-treated dermis. Fibrils in cross sectional view are compared. Scale bar, 100 nm.

Triton-treated dermis, which mechanically corresponds to S_b_ of the whole dermis, was similar to S_b_ in the cross sectional shape of fibrils (Fig 9B), in the cross-bridge number (Fig 6B), in the fibril diameter (Fig 7B) and its distribution pattern (Fig 4B), and in the inter-fibril distance (Fig 8B). The averages of the corresponding parameters in the Triton-treated dermis and those in S_b_ were not statistically different. The cross section of the Triton-FT dermis, which mechanically corresponds to S_a_ of the whole dermis, is shown in Fig 9A. In the Triton-FT dermis the number of cross-bridges decreased to a level similar to that of S_a_ (Fig 6B). The average of the cross-bridge number of Triton-FT dermis was not statistically different from that of S_a_. In Triton-FT dermis a small but statistically significant increase in the fibril diameter (Fig 7B) and a large increase in the inter-fibril distance (Fig 8B) were observed when compared with those of Triton-treated dermis.

### Suspension of Collagen Fibrils

**Fig 10:**
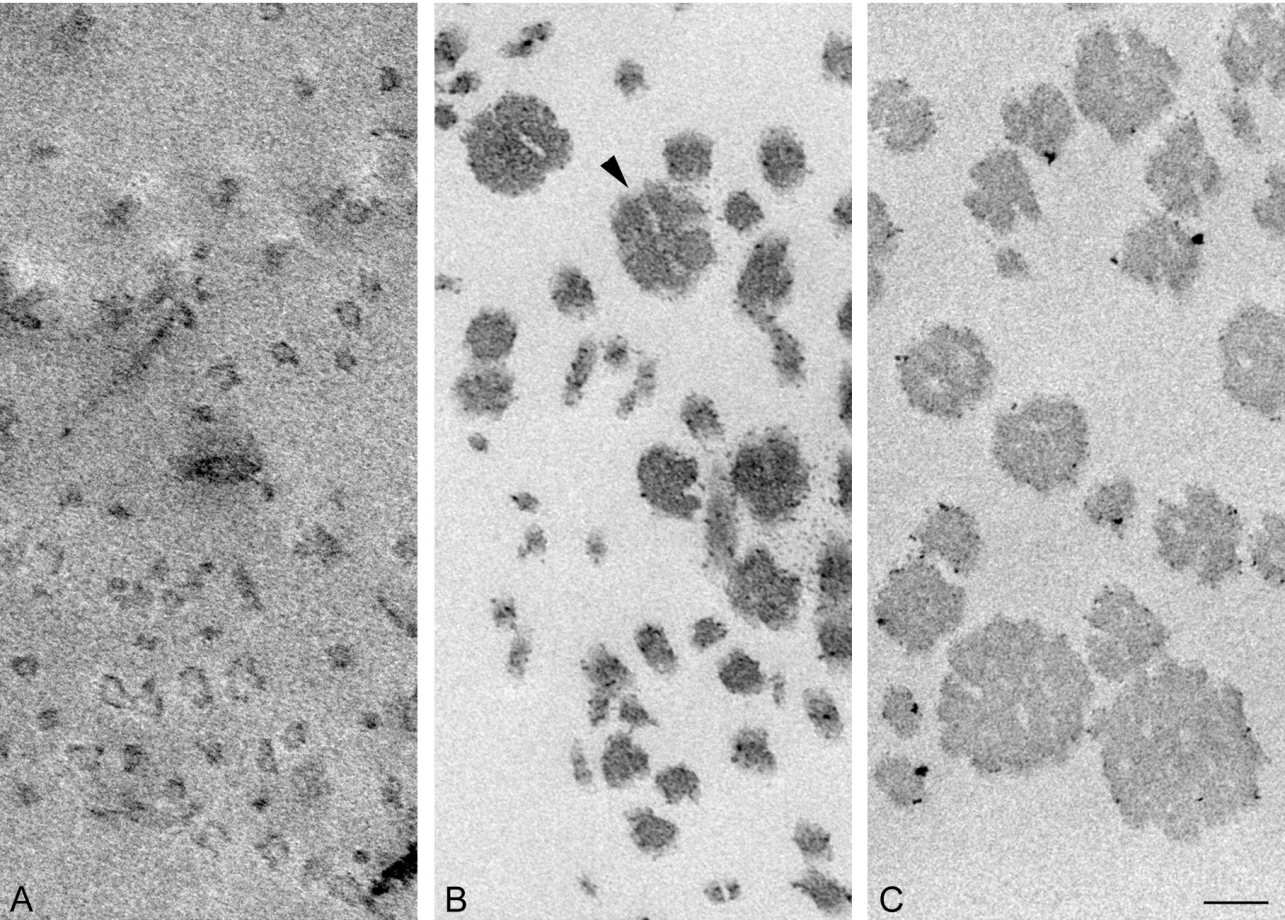
Electron micrographs of collagen-fibril suspensions. Samples were aggregated by tensilin (C), which were then dispersed either by FT treatment (A) or by softenin (B). The arrowhead indicates a fibril that appears as if 4 or more thinner fibrils were embedded in a less electron-dense matrix to form a fibril. Scale bar, 100 nm.

Tensilin aggregated the isolated collagen suspension, and such aggregates were dispersed by the addition of softenin or by the FT-treatment as was reported previously [3], [7]. The electron micrographs of the aggregated or dispersed samples are shown in Fig 10. In the aggregates, the cross sections of fibrils had a similar appearance to those in the whole dermis. There were two differences though. One was the difference in the diameter of fibrils. The range of the diameter distribution in the aggregates was 41–196 nm; fibrils with less than 40 nm in diameter, which were the major components in the dermis, were never observed in the suspensions (Fig 4C). The mean diameter of fibrils in the aggregates was 121 ± 40.7 nm (average ± S.D., n = 30), which was more than twice the mean of those in the dermis. The other difference was the absence of cross-bridges: they were never observed in suspensions irrespective of whether they were aggregated or dispersed. Electron-dense spots, however, were sometimes observed on the peripheries of fibrils as in the dermis.

Thinner fibrils prevailed in the dispersed samples (Fig 4C) especially in the FT samples, in which all fibril diameters were less than 52 nm and 10–30 nm fibrils represented about two thirds of all fibrils. In the softenin-dispersed samples also the fibrils with diameter less than 52 nm prevailed although fibrils with a diameter more than 100 nm were observed. Some thick fibrils of softenin-dispersed samples showed the internal structure that appeared as if several thin subfibrils had been embedded in a matrix of lower electron density (Fig 10B). The statistics on the fibril diameter showed that the differences between the three treatments were significant (Fig 7C).

## Discussion

The present study clearly showed ultrastructural differences in collagen fibrils that corresponded to the differences in the three mechanical states of catch connective tissues. The structural differences observed involved the number of cross-bridges between fibrils, the distance between fibrils, and the diameter of fibrils.

### Cross-Bridges

Collagen fibrils had protruding arms that sometimes made cross-bridges between adjacent fibrils. The number of cross-bridges increased with dermal stiffness, which strongly suggests that these cross-bridges provide passive resistive forces against shearing forces between fibrils, perhaps by opposing rotation around the sites to which the arms attached and extension along the arm axis.

The arms protruded repetitively along the fibril axis with a similar repeat period to the D period of collagen fibrils. The locations from which arms protruded seem to correspond to a particular zone of the cross-striation repeat pattern. The D-repeat patterns of the adjacent fibrils were usually aligned and in such cases the cross-bridges and arms protruded perpendicularly to the fibril axis, and in other cases the cross-bridges were slanted. These observations suggest that both the “origin” zone from which arms protruded and the “insertion” zone on the adjacent fibril to which arms attached were determined in the D-periodic striation. The origin zone and the insertion zone seemed to be in a similar position in the D-periodic striation. The observation that D-repeat patterns of adjacent fibrils were usually aligned suggests that the aligned disposition was mechanically least strained, which may support the notion that cross-bridges provide passive resistive forces against the shearing forces between fibrils.

Cross-bridges were observed in both the whole dermis and the Triton-treated samples, which showed that cross-bridges were resistant to the detergent. The number of cross-bridges in Triton-treated dermis was similar to that of the dermis in S_b_. In contrast, in the Triton-FT dermis a large decrease in the number of cross-bridges was observed. The crystals of ice formed between fibrils at freezing probably broke cross-bridges through pulling fibrils apart, which was also suggested from the increased inter-fibril distance in Triton-FT dermis. The Triton-treated dermis and the Triton-FT dermis mechanically correspond to the dermis in S_b_ and to that in S_a_, respectively [3]. The reduction in the cross-bridge numbers that occurred in softening both in the whole dermis and in the dermis in which cell membranes were disrupted again supports the notion that cross-bridges provide resistive forces.

Arms were not observed in the isolated collagen suspensions. This suggests that the arms either detached or changed conformation during the isolation procedure that used guanidine-HCl and trypsin. The result that tensilin aggregated the fibrils devoid of arms showed that the effect of tensilin was not related to the arms, which strongly suggests that tensilin was not a component that constructed arms.

In the 70-nm-thick sections, the average number of cross-bridges radiating out from a fibril in the standard state dermis was 0.46 and the longest cross-bridge observed was 67 nm. As the length of the isolated collagen fibrils of sea cucumbers is about 200 μm [12], a fibril might have ca. 1300 bridges along its axis. Such large numbers of cross-bridges suggests that all the fibrils not more than 67 nm apart from the nearest neighbors were cross-bridged to make a bundle that possibly corresponds to a fiber observed in optical microscopes [11]. The cross-bridges possibly made the bundle both thicker and longer because the fibrils are partly overlapping and cross-bridged at their ends; this arrangement would make the bundle longer than the fibrils themselves. The mechanism for producing longer bundles probably facilitated a constitutive increase in dermal stiffness, especially if they became long enough to be continuous from one end of the tissue to the other end, which will be discussed in the last section.

The cross-bridges between collagen fibrils have been reported not only in sea-cucumber dermis [11] but also in sea-urchin, feather star and brittle star ligaments with catch properties [13–17] which suggests that the cross-bridging between collagen fibrils is one of the general stiffening mechanisms in catch connective tissues. Although the cross-bridges have been reported previously, the present work is the first to show the changes in cross-bridge numbers that corresponded to the differences in mechanical properties.

### Distance between Collagen Fibrils

The distance between fibrils decreased in the transition S_b_→S_c_. Such a decrease was, however, not observed in S_a_→S_b_. These results are in accord with the report that the tissue volume reduced in S_b_→S_c_, which was associated with the water exudation, but such a reduction was not observed in S_a_→S_b_ [5]. The tissue shrinkage very probably decreased the distance between fibrils, which was what we observed in the present study. Tamori et al. (2010) regarded the dermis as a hydrogel of macromolecules whose mechanical properties were determined by weak non-covalent bonds between the macromolecules [5]. The candidates for such bonds are hydrophobic, ionic, van der Waals, and hydrogen bonds. The formation of any bonds possibly causes water exudation and the bonds make the interfibrillar matrix stiffer and more viscous, which causes higher tissue stiffness and lower energy dissipation.

The bonds, of course, have their own stiffening effects, but in addition to that, the bond formation accompanying the closer fibril distance and dermal shrinkage may cause an increase in stiffness through the increase both in the volume fraction of fibrils and in the load transfer parameter, which will be discussed in the last section.

The closer fibril distance may also contribute to tissue stiffening through increasing the probability of making cross-bridges. This mechanism has been proposed as the stiffening mechanism in the sea-urchin compass depressor ligament (CDL) [14], [18]. Ribeiro et al. (2011, 2012) studied ultrastructural differences of CDL not excised from the test, in which organs including the lantern were not removed [14], [18]. They applied acetylcholine to the CDL to induce S_c_, which caused stretching of the ligament along the longitudinal axis due to the lantern movement that was also induced by acetylcholine. They found a decrease in the distance between fibrils in S_c_, which they attributed to gross morphological change, that is, the decrease in width of the ligament associated with passive stretching in length.

The relation between the fibril distance and the mechanical properties has been studied mainly in vertebrate connective tissue. Screen et al. (2006) reported that the incubation of rat tail tendon in phosphate buffered saline caused swelling of its matrix and lead to a reduction in the maximum modulus [19]; the swelling seems to be the direct cause of softening. In sea cucumber dermis, however, the gain or loss of water is not the direct cause of the stiffness changes [5].

The stress-strain curves of the dermis showed prominent toe regions in S_a_ and S_b_ but not in S_c_. This difference can be interpreted as a decrease in the interfibrillar distance in S_c_. Collagen fibrils make a three dimensional meshwork in the dermis [11]. When the dermis is stretched, the fibrils first rotate to align in the direction of the stretch, which corresponds to the toe region, and the further stretch causes the stretching of fibrils, which corresponds to the linear region of the stress-strain curve [6]. The closer fibril distance in S_c_ implies a smaller mesh size. A smaller strain is enough to align the fibrils in a smaller mesh and thus the toe region becomes smaller in the stiff dermis.

### Collagen Fibril Diameter

The fibril diameter was a little larger in stiffer states. The changes in fibril diameter were likely caused by the lateral fusion and disassembly of fibrils, which was strongly suggested by the diameter-distribution patterns in the isolated collagen suspensions. Fibrils in the aggregated suspensions had larger diameters compared with those in the dissociated suspensions. The diameter distributions of these two kinds of suspensions had only a narrow overlap region. Because the dissociated samples were prepared from the tensilin-aggregated samples, the thinner fibrils in the former were produced from the thicker fibrils in the latter through the disassembly of the thicker fibrils by FT treatment and by softenin. This suggests the reverse process also occurred: the aggregates produced by tensilin were formed through the lateral fusion of thinner fibrils. In softenin-dispersed suspensions some thick fibrils showed an internal structure that appeared as if several thin sub-fibrils were embedded in a matrix of less electron density, which suggests that a fibril was made of several subfibrils packed loosely together. The aggregation caused by tensilin has been interpreted as the result of an increase in the cohesive forces between collagen fibrils [7], although the cohesive forces have not been measured. Our unpublished observations described below strongly suggested the changes in cohesive forces. The suspension of collagen fibrils made aggregates when tensilin was added to the suspension, and thus formed aggregates disintegrated into smaller aggregates when softenin was added. Such smaller aggregates in softenin were quite susceptible to gentle currents given by a pipet; they fell apart into much smaller aggregates or dissolved away. The aggregates formed in the suspension containing tensilin alone were more resistant to pipetting. The microscopical observations of softenin-treated samples needed special care not to give shearing forces when we put a cover slip on a drop of suspension containing aggregates on a slide glass. Otherwise the aggregates dissolved into suspensions. The aggregates formed by tensilin alone were more resistant to shearing. Both pipetting and shearing from a cover slip could be regarded as simple mechanical tests in which shearing forces were imposed on the aggregates to see how big were the resistive cohesive forces against shearing. Our observations strongly suggested that tensilin increased the cohesive forces between collagen fibrils that constituted the aggregates. Increased cohesive forces probably caused the lateral fusion of fibrils and also caused tighter packing of subfibrils in a fibril. The lateral fusion could have made fibrils both thicker and longer, facilitating the entanglement of fibrils to form aggregates. It is likely that lateral fusion through their increased cohesive forces occurred both in collagen suspensions and the dermis at the transition S_a_→S_b_ because tensilin causes this transition in the tissue.

Erlinger et al. (1993) proposed a similar mechanism [20]. In the collagenous crinoid ligaments they found fibrils much thinner than those of collagen. The authors regarded them as the “protofibrils” of collagen and proposed that the reversible disaggregation of collagen fibrils may responsible for the stiffness changes. Such fibrils with small diameters have been observed in other catch connective tissues and were described under various names such as microfibrils in echinoids [21] and 8 nm filaments in ophiuroids [17]. Birenheide and Motokawa (1994) observed microfibrils of crinoid ligaments under non-stretched condition and under stretched condition [22]. From the comparison of the staining properties of the banding patterns the authors concluded that the microfibrils were not collagen but very probably fibrillin.

Collagen fibrils both in suspensions and in the dermis had irregular perimeters in cross section and some thick fibrils sometimes had internal electron-lucent areas. These features have been reported in the dermis of other sea cucumbers [11], [12], [23]. The irregular humps on the perimeters also suggest that fibrils in the dermis, just like those in suspensions, are made of several subfibrils laterally packed together but not very tightly. For example, a fibril with a perimeter deeply folded into the center of the fibril, making its cross sectional shape like a four-leaved clover, is very likely made of four thinner fibrils stuck together only at the center of the aggregate. The electron-lucent areas in fibrils could be interpreted also by less tight packing: the subfibrils probably have varied length [12] and are aligned with their ends staggered; if such subfibrils are loosely packed, some spaces are formed inside the subfibril bundles, which will appear as a hollow space in cross sections. It is expected in such loose bundles that their ends are not completely filled with subfibirls, which will also appear as though the fibrils contained spaces in cross sections. In the latter situation the electron-lucent area is continuous with the outside matrix space. It is thus not surprising that electron-dense spots, similar to those found on the fibril-perimeters facing towards the spaces outside of the fibril, were sometimes found on the contour of the electron-lucent areas, these areas being, according to the present interpretation, continuous with the space outside the fibril.

Tensilin is a protein that invokes the transition S_a_→S_b_, and softenin produces the reverse transition S_b_→S_a_; the FT treatment is a more potent softener than softenin in the latter transition [3]. Thus the changes in cohesive forces caused by the action of these agents on the suspensions by these agents, which were reflected in the prominent changes in fibril diameter, would be expected to occur also in the dermis. Such prominent changes were, however, not observed in the dermis. The non-significant increase in the diameter did occur at S_a_→S_b_ in the whole dermis but the diameter even decreased a little in Triton-treated samples at S_a_→S_b_. This may be explained by possible antagonizing effects of cohesive forces on the fibril diameter: larger cohesive forces cause fibrils to fuse together to be thicker fibrils but they also draw the subfibrils nearer to make a tightly packed fibril and thus cause the decrease in the fibril diameter, both of which were observed or suggested in suspensions. The prominent diameter increase in suspensions suggests that the former effect was free to work in isolated collagen fibrils. In the dermis with other molecules such as proteins and proteoglycans associated with collagen fibrils, however, the cohesive forces were not free to increase the diameter, which was shown by the narrower fibril-diameter distribution of the dermis compared to that in suspensions. It is thus likely in the dermis that the diameter-increasing effect of cohesive forces was mostly cancelled by the decreasing effect, which resulted in the non-significant increase in diameter. Although the diameter increase was non-significant in the dermis, the increase in the cohesive forces was likely as large as that occurring in suspensions. The increase in the cohesive forces between subfibrils comprising each fibril increases the elastic stiffness of the fibrils and thus increases the elastic stiffness of the tissue as will be discussed in the last section.

Freeze-thawing (FT) caused a small but statistically significant increase in the fibril diameter in Triton-FT dermis, while it caused a large decrease in suspensions. It has been discussed that FT treatment broke cross-bridges, possibly by pulling the fibrils apart when ice was formed during freezing. Ice formation likely also pulled subfibrils apart, which caused fibrils to fall apart into thinner fibrils in suspensions. In Triton-FT dermis, in which collagen fibrils are associated with other molecules, however, such molecules possibly prevented the fibril from falling apart and only the increase in the distance between subfibrils occurred, which caused the fibril-diameter increase in Triton-FT dermis as we observed.

Stretch softening is a feature characteristic of the soft state: stiffness decreases as the dermis is stretched by more than 10%. This feature is explainable from the decrease in the cohesive forces between subfibrils. In a fibril in which cohesive forces are low, tensile strain likely causes slip between subfibrils. They are pulled out of fibrils as tensile strain becomes larger and eventually the fibrils fall apart, which causes a drastic decrease in stiffness of the dermis. Slip also accounts for the large energy dissipation in the soft state.

The D period of the dermis was the same irrespective of mechanical state. Barbaglio et al. (2015) reported a decrease in the D period in CDL of sea urchins when they were soft [24]. However, in peristomial membranes, another catch connective tissue in the same sea urchin, the authors observed no changes in the D period. They attributed the decrease in the D period to de-stretching of the CDL. Hidaka and Takahashi (1983) found no difference in the D period in the sea-urchin spinal ligaments when fixed in different ligament length or when the ligament was made soft by adrenaline [21]. These studies together with our present results indicate that the changes in the D period have little relevance to the mechanism of stiffness change in most catch connective tissues. The changes in D-period and fibril diameter have been reported in vertebrate connective tissues. Unlike the rapid changes found in catch connective tissues, the vertebrate collagenous tissues alter their stiffness only slowly: with age cross-links are formed between collagen fibers and the tissues become stiffer. Treatment with methylglyoxal, a naturally occurring metabolite known to form collagen cross-links associated with ageing, increased yield stress, D-period and fibril diameter in rat tail tendons [25].

### Nested Fiber-Reinforced Composite Model

The holothurian dermis can be regarded as a fiber-reinforced composite material in which fiber components with a high elastic modulus (E_f_) are embedded in a matrix of hydrogels with a low elastic modulus (E_m_) [6]. Various models have been developed to give the elastic modulus of fiber-reinforced composite materials (E_c_). The E_c_ of the simplest model in which fibers lie parallel to the externally applied tensile force is given by the following equation [26]:

$$E_{c}=\eta E_{f}V_{f}+(1-V_{f})E_{m}$$ (1)

where V_f_ is the volume fraction of the fiber component. The length correction factor η is 1 when each fiber is continuous from one end of the composite to the other end; otherwise η takes a positive value less than 1. The η is given by the following equation:

η=1−[tanh(βL/2)](βL/2) (2)

where L is the fiber length and β is the load transfer parameter given by the following equation:

$$\beta ={\left\{8G_{m}/[E_{f}\Phi^{2}\ln(D/\Phi )]\right\}}^{1/2}$$ (3)

in which G_m_ is the shear modulus of the matrix; D is the mean distance between fibers; and Ф is the radius of the fiber. From Eq 1, higher E_c_ is expected when fibers are stiffer (higher E_f_) and occupy larger volumes (higher V_f_), and when the matrix is stiffer (higher E_m_) and when η is larger, which is valid for more complex models. Eq 2 tells us that larger η is expected when fibers are longer (larger L) and β is larger. From Eq 3 the parameter β is expected to become larger when the matrix is stiffer (larger G_m_) and the fibers are placed nearer to each other (smaller D).

**Fig 11:**
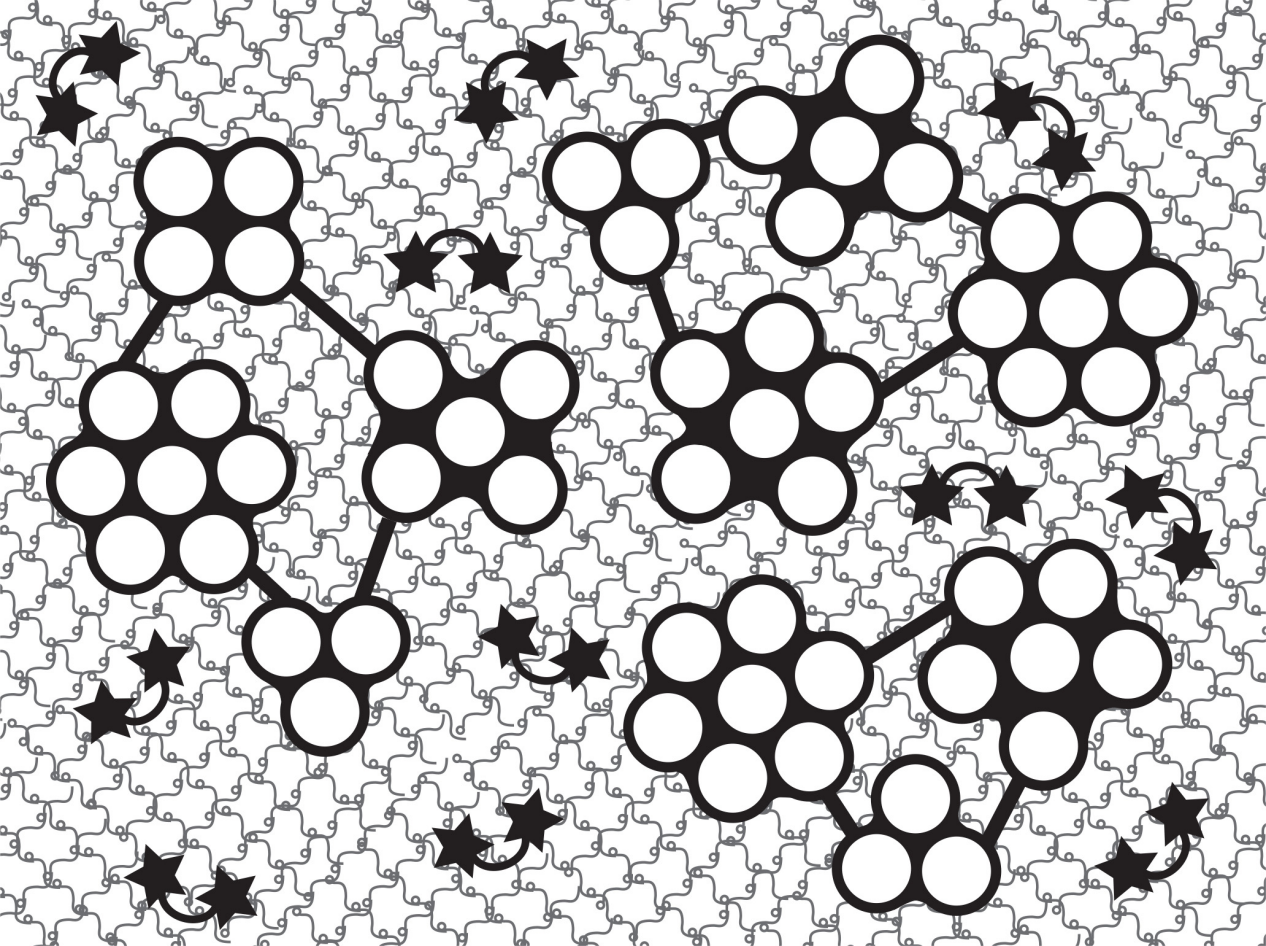
Nested fiber-reinforced composite model. The holothurian dermis is modeled as a material with 3 levels each of which could be regarded as a fiber-reinforced composite. The first level: collagen fibrils are made up of subfibrils (white circles) embedded in a matrix, shown by black that fills between subfibrils; the stiffness of the matrix is controlled through tensilin and softenin. The second level (bundle level): fibrils are cross-bridged by arms (black bars) to be a bundle that is embedded in a matrix whose stiffness is regarded here to be controlled by the number of cross-bridges. The third level (dermis level): the bundles are embedded in a matrix made of a hydrogel of proteoglycans whose stiffness is controlled by bonds between proteoglycans; bond formation is associated with water exudation. Proteoglycan molecules are drawn here as tadpole-shaped ones with a short stranded tail that fabricated the orthogonal network [26]. The arcs with a star at each end denote bond between proteoglycans.

The present results suggest that the sea cucumber dermis could be regarded as a nested fiber-reinforced composite with three levels (Fig 11): the first level is that of the fibrils; the second level is that of the fibril bundles; and the third level is that of the whole dermis. The parameters such as E and V of each level are denoted by the subscripts 1, 2 and 3 here.

At the first level, a fibril with the elastic modulus E_c1_ could be regarded as a composite of subfibrils with the elastic modulus E_f1_ as a fiber component embedded in a matrix with the elastic modulus E_m1_. The present results suggest that in the transition S_a_→S_b_ tensilin increased E_c1_ by increasing E_m1_, and by the increase in η_1_ through the increase in β_1_ that was produced by the increase in G_m1_ and the decrease in D_1_. The decrease in D_1_ was suggested from the loose packing in the softenin dissociated suspension.

At the second level, the fibril bundle with the elastic modulus E_c2_ could be regarded as a composite of fibrils with the elastic modulus E_f2_ (= E_c1_) embedded in a matrix of the elastic modulus E_m2_. The cross-bridge formation between fibrils could be regarded as the stiffening of the matrix between fibrils; cross-bridges contribute to the increase in E_c2_ by increasing E_m2_ and by increasing the η_2_ that was produced by the increase in β_2_ through the increase in G_m2_. The cross-bridges probably increase L_2_, which contributes to the increase in η_2_.

At the third level, the dermis with E_c3_ could be regarded as a composite of fibril bundles with E_f3_ (= E_c2_) embedded in a matrix of a proteoglycan hydrogel with E_m3_. In Fig 11, we assumed that the matrix of the present sea cucumber dermis was made of networks of orthogonally fabricated proteoglycans of short length after Scott (1988) [27]. The bond formation associated with water exudation at S_b_→S_c_ very likely increases E_m3_ and G_m3_ possibly through bonding between proteoglycan molecules. The η also increases in S_b_→S_c_ through a decrease in D. We observed the decrease in the fibril distance D_2_; the decrease in D_3_ and perhaps D_1_ is also expected from the tissue shrinkage at S_b_→S_c_. If the bond formation occurred mainly in the matrix embedding the fibril bundles, the decrease in D_3_ is expected to be greater than those of D_2_ and D_1_, which increases the volume fraction V_f3_. The bonds might also be formed between collagen fibrils and matrix proteoglycans, which increases the load transfer length.

We interpreted our results by the model that assumed the aligned fibrils. Such alignment occurs when sea cucumbers experience large deformation that occurs in both situations in which extremely stiff or extremely soft dermis is needed. The need of stiff dermis is found when sea cucumbers were subjected to large forces imposed by rush current surges or when they were bitten and pulled by potential predators. The need of soft dermis is found when sea cucumbers pass through the narrow crevices to hide inside rocks to protect themselves under the stormy weather and when they autotomize or reproduce by fission. Because these situations are vital to sea cucumbers we modeled the dermis with aligned fibrils only. What we found in the present study is of course relevant to the stiffness changes of the dermis under small deformations in which fibrils are not aligned. The thicker fibrils after fusion and the fibrils connected to larger number of adjacent fibrils by cross-bridges have higher bending stiffness and higher resistance against the movement through the matrix in the process of reorientation under imposed strain, and thus both of them contribute to the dermal stiffness.

In the present model we hypothesized that the changes in stiffness of the matrix occurred only in the transition S_b_→S_c_. Of course there remains possibility that changes in stiffness of the matrix also occurred in the transition S_a_→S_b_, on which we have no information so far.

In short, we suggest that tensilin made collagen fibrils stronger and stiffer in S_a_→S_b_; cross-bridging by arms caused the fibrils to be a continuous network of bundles both in S_a_→S_b_ and in S_b_→S_c_; the matrix embedding the fibril network became stiffer in S_b_→S_c_ that was produced by bonding associated with water exudation. The molecular mechanisms involved in S_a_→S_b_ and in S_b_→S_c_ have been believed to be different because of the presence of specific agents such as tensilin and NSF responsible for the respective stiffening processes. The present study verified this notion in part but revealed that cross-bridging is another mechanism common to both processes. The knowledge of what molecules construct arms and how they work in cross-bridging are indispensable to the understanding of the mechanism of connective tissue catch. So far, the biochemical nature of the arms is poorly understood but histochemical studies suggested that certain structures that appeared to bind to collagen fibrils contain proteoglycans. Such structures have been observed by ruthenium red staining in ophiuroid ligaments [17] and by Cupromeronic Blue staining in holothurian dermis and crinoid ligaments [20, 27]. Ultrastructural studies with stains specific to proteoglycans on the holothurian dermis at three different mechanical states will provide further information on the nature of cross-bridges.
